# A Role for Postoperative Negative Pressure Wound Therapy in Multitissue Hand Injuries

**DOI:** 10.1155/2018/3629643

**Published:** 2018-03-26

**Authors:** Hyung Sup Shim, Ji Seon Choi, Sang Wha Kim

**Affiliations:** ^1^Department of Plastic and Reconstructive Surgery, College of Medicine, The Catholic University of Korea, St. Vincent's Hospital, Seoul, Republic of Korea; ^2^Department of Plastic and Reconstructive Surgery, College of Medicine, Seoul National University Hospital, Seoul National University, Seoul, Republic of Korea

## Abstract

In this study, we compared outcomes in patients with acute hand injury, who were managed with or without negative pressure wound therapy (NPWT) after reconstructive surgery. All of the patients who sustained acute and multitissue injuries of the hand were identified. After reconstructive surgery, a conventional dressing was applied in Group 1 and NPWT was applied in Group 2. The dressing and NPWT were changed every 3 days. The mean age and Hand Injury Severity Scoring System score of both groups were not significantly different. Disabilities of the Arm, Shoulder, and Hand (DASH) scores were evaluated 1 month after all the sutures were removed and 1 year postoperatively, which were both significantly lower in Group 2. Applying NPWT to the hand promoted wound healing by reducing edema, stabilizing the wound, and providing immobilization in a functional position. Early wound healing and decreased complications enabled early rehabilitation, which led to successful functional recovery, both objectively and subjectively.

## 1. Introduction

A significant proportion of hand injury cases are multiple faceted and heavily contaminated and involve composite soft tissue and bone injuries due to the complexity of the anatomy and function of the hand. As a result, hand injuries are often difficult to manage promptly and require multiple staged serial treatment. On the other hand, functional recovery is as important as structural reconstruction and resurfacing in hand injuries, as the hand is a functional unit. Early exercise and rehabilitation improve functional recovery; therefore, wound healing should be achieved as soon as possible [1–3]. Negative pressure wound therapy (NPWT) is a good alternative not only for management during the preoperative period of early reconstruction, but also for early recovery after reconstruction.

NPWT has been widely used for almost every type of wound, from acute traumatic wounds to chronic intractable wounds [4]. It generates a subatmospheric pressure of 50−150 mmHg in either a continuous or an intermittent mode [2]. Although the exact mechanism is undefined, the effects of NPWT are to remove excess fluid and debris, improve tissue perfusion, and promote wound healing by enhancing formation of granulation tissue and decreasing the size of the wounds [4–8].

In this study, we compared outcomes in patients with acute hand injury who were managed with or without NPWT after reconstructive surgery.

## 2. Materials and Methods

This study was approved by the Institutional Review Board. All of the data were analyzed anonymously and according to the principles in the 1975 Declaration of Helsinki, revised in 2008.

The study was a prospective open trial. All of the adult patients (>20 years) who sustained acute multitissue injury of the hand from January 2013 to December 2016 were enrolled with the following criteria. The patients included in this study sustained acute hand injury of a similar severity, as assessed by a Hand Injury Severity Scoring System (HISS) score of 21−50 (Table 1), which is defined as a moderate severity level II injury, and underwent reconstruction within 3 days after injury by two surgeons. Patients with a medical history of impaired motor function, injury to the peripheral nerves and/or vessels distal to the wrist, or a bone fracture requiring transarticular fixation with a Kirchner (K) wire, a congenital hand deformity, an operation history on the same hand, and underlying diseases including autoimmune diseases such as rheumatoid arthritis or systemic lupus erythematosus or those taking medications that could influence wound healing were excluded from the study. Informed consent was obtained from patients who met the inclusion criteria before randomization. Patients were randomly assigned to the control or experimental group following a simple randomization procedure (computerized random numbers) achieved using opaque envelopes.

Reconstruction was performed according to the injury on a case-by-case basis. Bone fractures including fractures of the phalangeal, metacarpal, and carpal bones were fixed with K-wires averting articular surfaces, and the tip of the K-wire was closely cut and embedded under the skin. Open reduction and ligament repair were performed as required for dislocated joints. Tendons were repaired accordingly for tendon rupture or avulsion injuries. The skin lacerations were closed primarily, and skin and soft tissue defects were reconstructed with local flaps or a skin graft. A silastic drain was inserted before closure.

After reconstruction, a conventional dressing was applied over the closed skin using polyurethane foam with a compressible elastic bandage, and a short arm splint was applied in a functional position in Group 1 (control group). By contrast, NPWT (CuraVAC®, CGBio, Seongnam-si, Gyeonggi-do, Korea) was applied at a pressure of 75 mmHg in continuous mode in Group 2 (experimental group). The secondary dressing for Group 2, including Vaseline gauze, was applied before NPWT. The dressing and NPWT were changed every 3 days. In both groups, when the skin was completely healed within 2 weeks after the injury, the dressing or NPWT was removed, followed by the sutures. Physical therapy was started under consultation with the rehabilitation medicine department after wound healing, and the allocation information to each group was not provided to reduce bias. Physical therapy was performed twice weekly for 4 weeks with individual home-exercise instruction.

Data were collected from the patient's medical records and radiographs. The baseline characteristics collected were age, sex, date of injury, injury site, and the HISS score.

Time to recover over 90% of the full range of motion (ROM) compared to the normal values of full flexion and extension was analyzed for every interphalangeal and metacarpal joint. In addition, the Disability of the Arm, Shoulder, and Hand (DASH) score was evaluated 1 month after suture removal, when the skin was completely healed and 1 year postoperatively.

Evaluated complications were hematoma, infection, wound disruption, or a secondary operation.

Comparisons between the two groups were performed using chi-square test and Fisher's exact test. A *p* value < 0.05 was considered statistically significant.

## 3. Results

We identified 51 patients (17 females and 34 males; age: from 21–61 years; mean age: 39.8 years) with acute hand injuries who met the study inclusion criteria. A total of 21 patients received conventional dressing using polyurethane foam and a short arm splint, and 30 patients received NPWT. The mean age of Group 1 was 41.4 (range: 22–61) years and that of Group 2 was 39.9 (range: 21–61) years. The mean HISS score of Group 1 was 33.6 (range: 21–50) and that of Group 2 was 35.7 (range, 21–50). No significant differences were observed in patient demographics or HISS scores between the two groups.

DASH scores were evaluated 1 month after all of the sutures were removed and 1 year postoperatively. The scores at 1 month averaged 33.14 (range: 18.3–48.3) in Group 1 and 22.67 (range: 5.8–40.1) in Group 2 (*p* = 0.031). The score at 1 year averaged 22.08 (range: 14.9–31.9) in Group 1 and 20.99 (range: 5.1–32.0) in Group 2 (*p* = 0.667). The hand joints recovered >90% of the full ROM at 46.9 (range: 30–61) days after injury in Group 1 and at 33.3 (range, 22–58) days in Group 2 (*p* = 0.022).

There were five complications: two hematomas and one infection were treated conservatively by drainage and antibiotics in Group 1, and two wound macerations in Group 2 healed conservatively without additional surgery. No difference in complications was observed between the two groups.

The statistical comparisons between the two groups are presented in Table 2.

**Figure 1:**
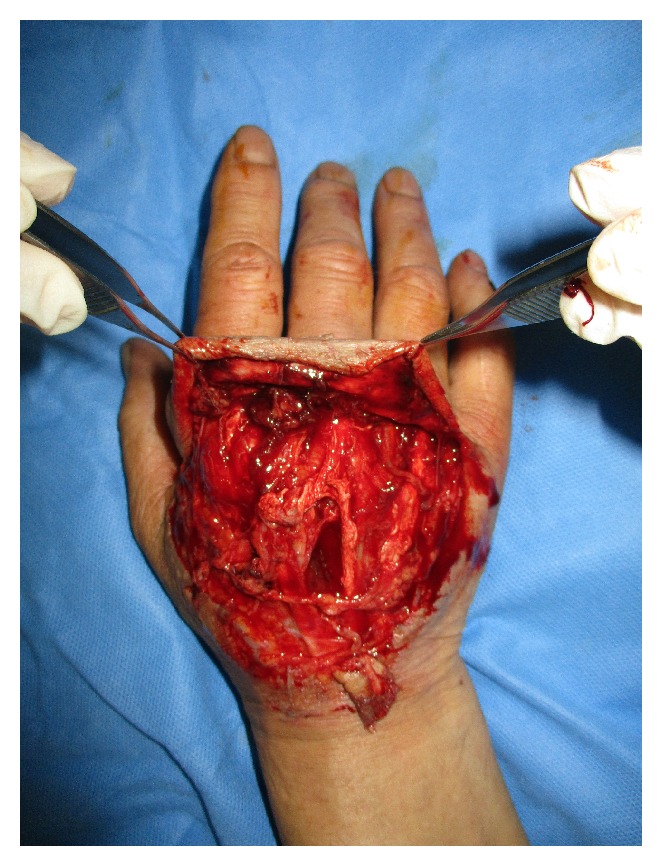
A 59-year-old male suffered multitissue injury of the right hand, including all of the dorsal skin and extensors.

**Figure 2:**
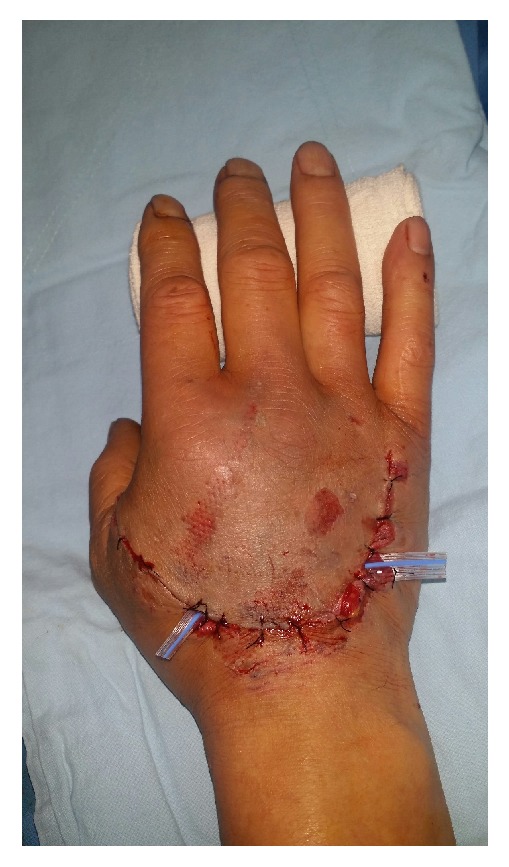
Emergency operations including tendon repair and wound closure were performed.

**Figure 3:**
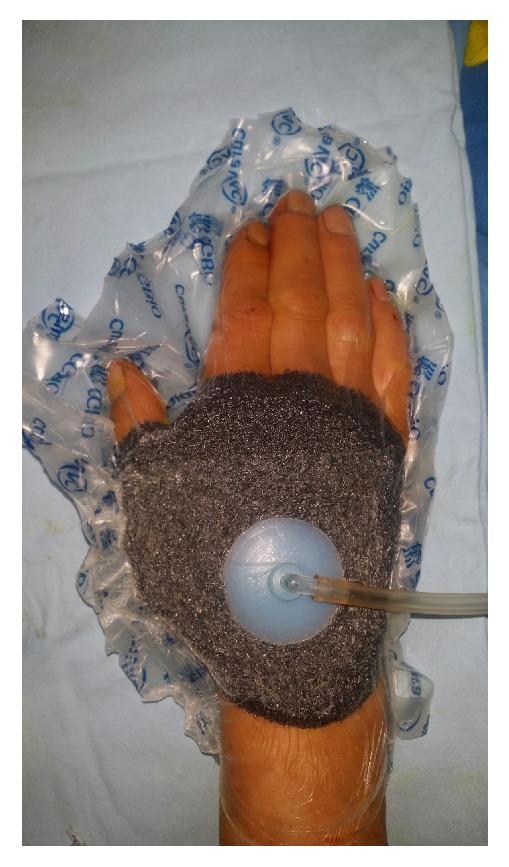
The wound was covered with negative pressure wound therapy (NPWT) immediately after surgery.

**Figure 4:**
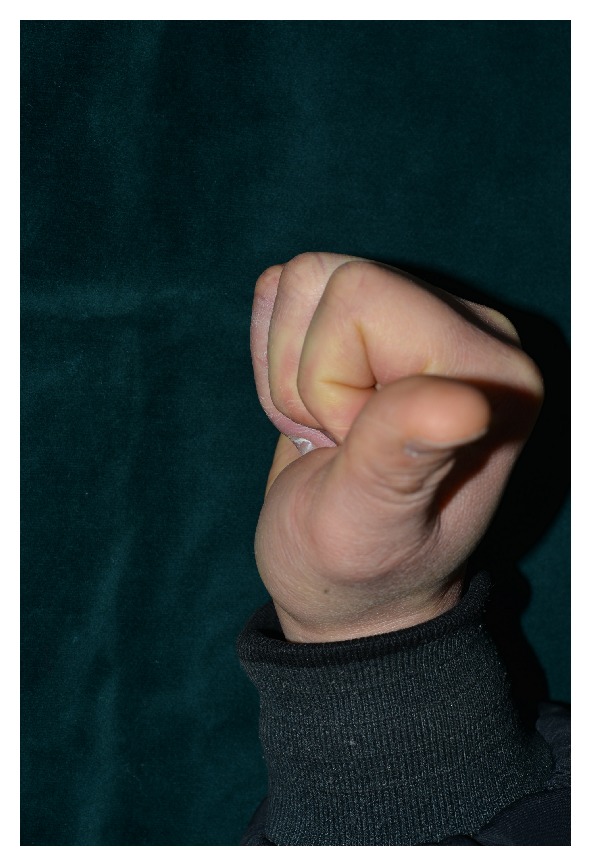
Full range of motion was achieved 4 weeks after surgery.

*Case 1. *A 59-year-old male visited the emergency room after his hand had been smashed in a heavy rolling machine. All of the dorsal skin on the hand was avulsed with multiple ruptures of the extensors (Figure 1). The ruptured second and third extensor digitorum communis and fifth extensor digitorum minimi were repaired, the avulsed skin envelope was tension- free repaired, and a silastic drain was inserted (Figure 2). Then the whole dorsal side of the hand except the fingers was covered with NPWT (Figure 3). NPWT was changed every 3 days. Full ROM was achieved without restriction of daily activity 4 weeks after suture removal (Figure 4).

**Figure 5:**
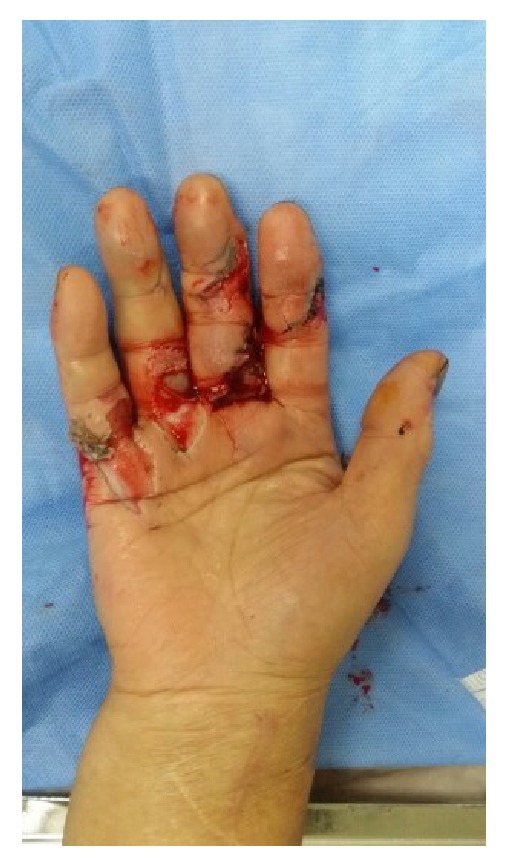
A 54-year-old male with multitissue injury of the right hand visited the emergency room. Flexors of the second and third fingers were ruptured, with an avulsion bone fracture and multiple skin defects.

**Figure 6:**
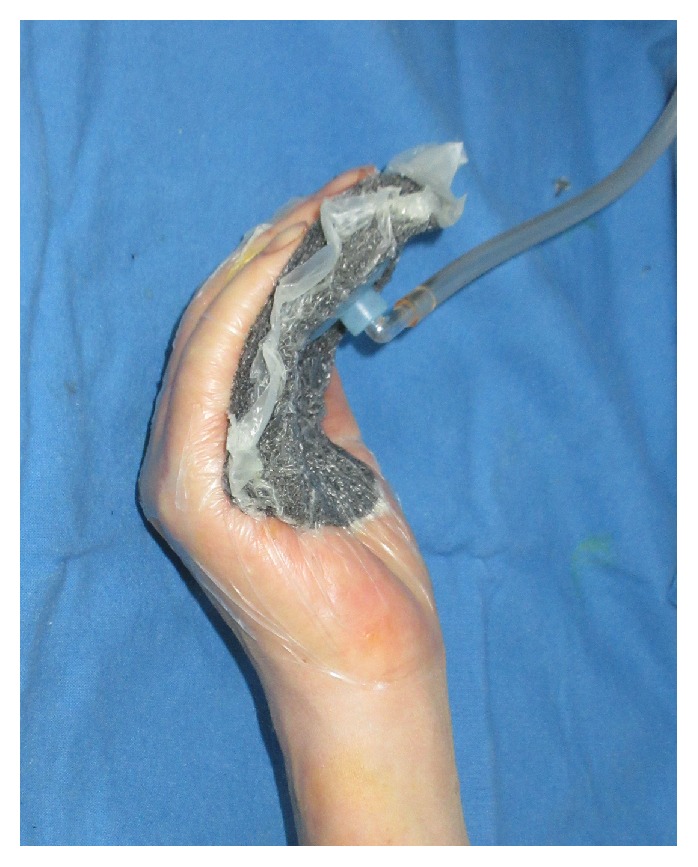
The patient was treated with negative pressure wound therapy (NPWT).

**Figure 7:**
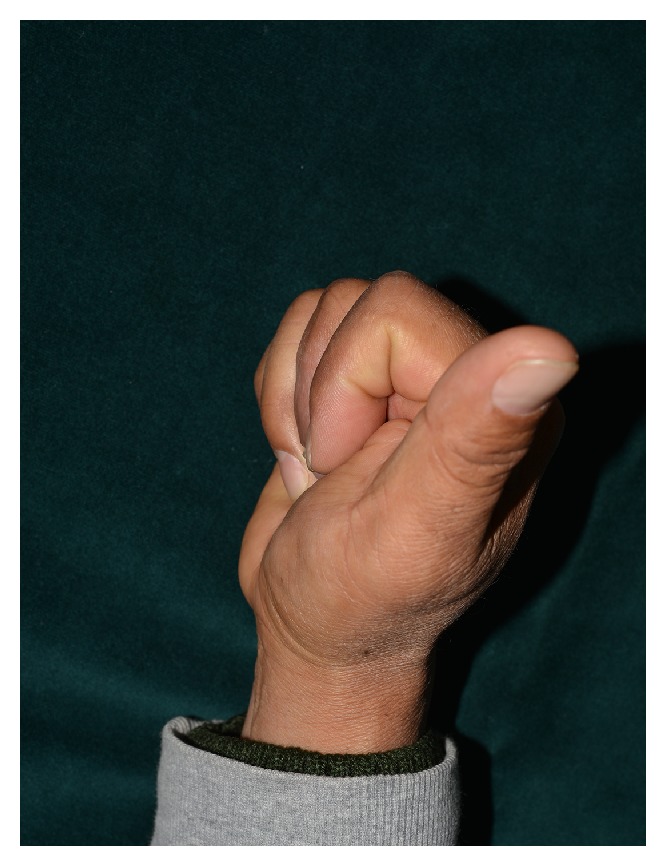
Full range of motion was achieved 2 months after surgery, without restriction of daily motion.

*Case 2. *A 54-year-old male suffered a multitissue injury of the right second to fifth fingers in a press machine accident. The third proximal phalangeal bone was fractured in an avulsed manner. The second and third flexor tendons were also ruptured, and multiple skin defects occurred on the volar side of the hand (Figure 5). The fractured bone was reduced while repairing the ruptured flexor tendons, and the lacerations and skin defects were repaired with skin grafts. Then, the whole volar side of the hand was covered with NPWT, which was changed every 3 days (Figure 6). The wound was healed 2 weeks later, and the sutures were removed. After 2 months of rehabilitation and physical therapy, the patient was able to use his hands freely with full flexion and extension and returned to work (Figure 7).

## 4. Discussion

NPWT was first reported in 1993 [9] and was introduced as “vacuum-assisted closure” for wound control and treatment by Morykwas et al. in 1997 [4, 10]. Since then, NPWT has been widely used not only for chronic nonhealing wounds, but also for acute traumatic injuries. Its effectiveness is thought to be due to decreased bacterial count, increased tissue perfusion, removal of exudates, and promotion of granulation tissue formation, all of which promote wound healing [4, 6, 10]. The NPWT system consists of foam connected to a vacuum pump through a connecting tube, and the whole system is covered with a semiocclusive dressing [7]. The application of NPWT has been expanded from managing and protecting the wound and preparing for final reconstruction to improving skin graft outcomes and patient comfort and thereby reducing cost [6, 7, 11, 12].

NPWT has been mostly used in patients undergoing hand surgery with soft tissue defects associated with trauma, burns, or infection [1–3, 13–15]. The effective use of NPWT in preparing soft tissue defects before reconstruction has been well described, and favorable results have been achieved in patients with bone, tendon, or nerve exposure [13–15]. On the other hand, use of NPWT after reconstruction has only been reported in selective cases. Most commonly, NPWT has been applied after skin grafting. NPWT stabilizes the graft and promotes adherence of the skin graft, which improves graft take [7].

The hand is a functional and mobile unit with a complex anatomy. Thus, reconstruction of hand injuries should focus not only on resurfacing with healthy soft tissue, but also on maintaining good muscle strength and flexibility of the tendons without adhesion [2]. Many joints of the hand require early rehabilitation of the ROM to prevent contracture. NPWT can be applied after reconstruction and offers several advantages. It can be used instead of conventional polyurethane foam and short arm splint dressings. NPWT simplifies the dressing while stabilizing the hand [1]. Use of NPWT splints of the hand in a functional position, and the hand can be molded into the desired functional position before applying suction [13]. The absence of a splint allows easy visualization of the position and status of the hand. Moreover, NPWT with foam allows only minimal motion of the joints, functioning as a sort of dynamic splint. NPWT as a partial dynamic splint has two advantages. One is that NPWT helps decrease swelling, which leads to better overall hand function. Hand wounds can remain swollen for some time following injury and become more swollen after reconstruction. Reduced swelling fosters early recovery of tissues, which leads to early rehabilitation. As in our cases, NPWT can also function as a negative drainage tool through the space using a silastic drain. A second advantage is that the minimal motion of the joint protects against severe contracture of the hand. These advantages explain the superior results of NPWT compared to a conventional dressing with a splint.

One of the limitations of using NPWT is that the pressure might compress the microvessels in the soft tissue, compromise the vascularity of the tissue, and decrease tissue perfusion. Applying NPWT could be of concern to surgeons, particularly in the hand, where blood circulation is limited to certain vessels and thin, pliable soft tissue with a weaker cushion effect. Some surgeons hesitate to use NPWT on the hands because of fear of restricted movement, difficulty of the application, and leakage due to the complex shape of the hand. Therefore, some modifications of commercially available NPWT have been reported, using gauze instead of a foam sponge or a sealing bag instead of semiocclusive dressing coverage [1–3, 13]. These modifications are suitable adjustments for hand injuries; however, they were reported as certain indicated cases and required surgical adjustment on a case-by-case basis.

The pressure applied through the NPWT foam evenly distributes the mechanical force to the wound. Morykwas et al. tested various suction pressures from 0 to 400 mmHg and found that 125 mmHg was optimal for increasing local blood flow [4, 7]. Current recommendations state that 50–150 mmHg of negative pressure is acceptable [13]. In our cases, the hand was placed in the most functional position, and the drain was connected to suction power and set to 75 mmHg. Although 125 mmHg is the standard pressure for NPWT [7], similar effects can be achieved at lower pressures [13, 16, 17]. In addition, some reports have demonstrated that tissue pressure increases beneath the NPWT in all types of wounds, is directly proportional to the amount of suction applied, and is most pronounced in circumferential dressing [18]. Previous authors have reported that increased pressure results in 17% decreased perfusion when circumferential NPWT is applied with a suction pressure of 125 mmHg [19]. The theories regarding the mechanism of action of NPWT suggest that compression of tissue decreases perfusion and concurrent hypoxia is a stimulus for angiogenesis. In addition, tissue hypoxia results in release of nitric oxide and local vasodilation [18, 20]. On the other hand, concerns regarding the safety of NPWT on tissues with compromised perfusion have also been raised [18, 19]. We are aware that there is a potential risk for perfusion from the compression effects of a circumferentially applied NPWT dressing on the hand. We did not find any evidence of reduced vascularity or compromised tissue perfusion as a result of using NPWT for hand injuries. By contrast, we noticed a significant reduction in edema. The compression provided by NPWT likely forces edema away from injured tissues. This ultimately results in decreased interstitial pressure, decreased compression of the vessels, and improved oxygen and nutrient supply. These results are likely to be the most important contributions of NPWT.

The HISS is the most commonly used measure to clinically assess hand injury severity [21]. It is evaluated by scoring the severity of each hand segment from skin, bones, motor function, and nerve injury. The total score is determined by adding the point values of hand injury severity, then classifying it according to the score obtained, expressed as grades I–IV [22]. In this study, we excluded patients with concomitant peripheral nerve injury because in this study, we compared the functional outcomes of patients with acute hand injury with or without NPWT after surgery, and nerve injury can interfere with the results of the functional outcome. In addition, we only included patients with hand injuries and HISS scores of 21–50, which is grade II, and those who underwent reconstruction within 2 weeks after the injury. Patients with HISS scores > 50 and who were severely injured are often difficult to manage and require several staged treatments that could not be performed within 2 weeks; therefore, they were excluded.

A quantitative assessment of hand dysfunction is much more difficult. Among the most commonly used scales is the DASH scale, a 30-item questionnaire that evaluates symptoms and physical function with a five-response option for each item. The DASH score is determined by calculating the circled responses. It produces a brief, self-administered measure of symptoms and functional status [23]. The only limitation is that the DASH is a subjective measurement that represents hand function but does not fully correlate with objective functional recovery. Therefore, we first evaluated the DASH scale to represent personal symptoms and subjective situations. Then, we also evaluated objective functional recovery by determining the period when recovery of ROM was >90%. A 90% recovery of ROM is almost full recovery of function, which enables daily activity, the end of rehabilitation, and the return to social life. In Group 2, the DASH scores were lower and the number of ROM recovery days was fewer compared to those in Group 1; these differences were statistically significant. Although the DASH score at postoperative 1 year was not different, the results suggest that NPWT was essential for early and fast recovery of hand function.

In the cases we described above, NPWT was successfully used to treat challenging hand injuries. Complications such as tissue loss, dehiscence, infection, or hematoma can have serious effects on the functional outcome. The use of NPWT on very thin skin flaps or over skin grafts, where there is a concern for hematoma, perfusion, and skin survival, was particularly useful. In addition, commercially available NPWT is increasingly evolving. The foam sponge has become thinner, more flexible, and customized to the defect; the connecting tube is slender and length-adjustable; and the vacuum pump system has become smaller and more easily portable. As NPWT maintains the injured hand in a stable state and can be changed every 3 days, most patients can be discharged and followed in the outpatient clinic, which is more convenient for patients and reduces hospital stay and costs.

In our experience of treating acute hand injures, NPWT is quick and easily applied. NPWT promotes wound healing by reducing edema, stabilizing the wound, and providing immobilization in a functional position. Early wound healing and decreased complications enabled early rehabilitation, which lead to a successful functional recovery, both objectively and subjectively.

## Figures and Tables

**Table 1:** Hand Injury Severity Scoring System (HISS).

	Score	Sum	Severity
Integumental injuries	0–40	<20	I: minute
Bone injuries	0–9	21–50	II: medium
Impairment of motor function	0–16	51–100	III: severe
Nerve injury	0–34	>100	IV: major

**Table 2:** Comparison between two groups.

	Group 1 (conventional dressing)	Group 2 (NPWT)	*p* value
Age (years)	41.38 ± 10.92	38.77 ± 1.68	0.375

HISS score	33.57 ± 1.86	35.73 ± 1.55	0.377

Time to recover over 90% of the ROM (days)	46.90 ± 2.05	33.30 ± 1.51	0.022

DASH score at one month	33.14 ± 1.68	22.67 ± 1.43	0.031

DASH score at one year	22.08 ± 2.03	20.99 ± 1.91	0.667

Complications	3	2	0.383
Hematoma	2	0	
Infection	1	0	
Wound disruption	0	2	

NPWT: negative pressure wound therapy; HISS: Hand Injury Severity Scoring System; ROM: range of motion; DASH: Disability of the Arm, Shoulder, and Hand questionnaire.
